# An approach to exploring associations between hospital structural measures and patient satisfaction by distance‐based analysis

**DOI:** 10.1186/s12913-020-06050-3

**Published:** 2021-01-13

**Authors:** Masumi Okuda, Akira Yasuda, Shusaku Tsumoto

**Affiliations:** 1 Nursing Department, Matsue Red Cross Hospital, 83-1 Horo-machi, 690-8506 Matsue, Shimane, Japan; 2 Department of Medical Informatics, School of Medicine, Shimane University, 89-1 Enya-cho, 693-8501 Izumo, Shimane, Japan

**Keywords:** Patient satisfaction, HCAHPS, Data mining, Clustering

## Abstract

**Background:**

Patient satisfaction studies have explored domains of patient satisfaction, the determinants of domains, and score differences of domains by patient/hospital structural measures but reports on the structure of patient satisfaction with respect to similarities among domains are scarce. This study is to explore by distance-based analysis whether similarities among patient-satisfaction domains are influenced by hospital structural measures, and to design a model evaluating relationships between the structure of patient satisfaction and hospital structural measures.

**Methods:**

The Hospital Consumer Assessment of Healthcare Providers and Systems 2012 survey scores and their structural measures from the Hospital Compare website reported adjusted percentages of scale for each hospital. Contingency tables of nine measures and their ratings were designed based on hospital structural measures, followed by three different distance-based analyses - clustering, correspondence analysis, and ordinal multidimensional scaling – for robustness to identify homogenous groups with respect to similarities.

**Results:**

Of 4,677 hospitals, 3,711 (79.3%) met the inclusion criteria and were analyzed. The measures were divided into three groups plus cleanliness. Certain combinations of these groups were shown to be dependent on hospital structural measures. High value ratings for communication and low value ratings for medication explanation, quietness and staff responsiveness were not influenced by hospital structural measures, but the varied-ratings domain group similarities, including items such as global evaluation and pain management, were affected by hospital structural measures.

**Conclusions:**

Distance-based analysis can reveal the hidden structure of patient satisfaction. This study suggests that hospital structural measures including hospital size, the ability to provide acute surgical treatment, and hospital interest in improving medical care quality are factors which may influence the structure of patient satisfaction.

## Background

Patient satisfaction is considered a key factor for improving health care quality. Medical care quality is commonly evaluated using questionnaires and interviews, followed by a variety of statistical analyses. Since the 1970s, domains of patient-satisfaction including global evaluation have been identified through factor analysis [1–3] to explore what domains consist of patient satisfaction as the structure of patient satisfaction, and relationships among domains are usually assessed by correlates [1–16] and regression analyses [4, 5, 9–18]. However, these traditional analyses are methodologically restricted when simultaneously analyzing the relationships of domains. With the growing interest in data mining, researchers have taken different approaches to questionnaires by applying distance-based analysis which analyzes domains simultaneously to evaluate the similarities among the domains [16, 19–22], which might be called the hidden structure of patient satisfaction.

There are three major methods using distances which may be applied to a questionnaire. Results are shown visually and are intuitively easy to understand, though their interpretations may be somewhat subjective. Most studies have applied one of these three methods: clustering, correspondence analysis or multiple dimensional scaling (MDS); however, they have not explored differences with respect to patient and hospital structural measures.

In our study, in what we believe to be a novel approach, we applied these three methods against a lack of objectivity to evaluate similarities among domains of patient satisfaction, and to explore whether similarities of patient-satisfaction domains differ by hospital structural measures. The methodological restrictions of traditional analyses are discussed first, followed by distance-based analysis.

Correlation analysis, introduced to patient-satisfaction studies in the 1960s [23], remains a major method of assessing relationships among patient-satisfaction domains [1–16]. The analysis requires normally distributed data and explores pairwise relationships, yielding results for each pair. Linear regression analysis, also requiring normally distributed data, has been used since the 1980s [4, 5, 11, 12, 14–18], and generalized regression analysis since the 1990s [9–11, 13], both in patient-satisfaction studies. Regression models investigate relationships between objective and exploratory variables, yielding as many results as the objectives. Score differences of domains insofar as they relate to patient and hospital structural measures are also explored domain-by-domain using inferential statistics, including chi-square tests, t-tests and analysis of variance [6, 8, 17]. Difficulties encountered in drawing conclusions from such results made inevitable the need to focus on selected measures, such as global evaluation and communication measures [4, 5, 14, 15, 24], not only because an overall rating is believed to represent the patients’ assessments, allowing for easier interpretation, but also because communication measures are one of the major determinants of global evaluation [6, 9, 25]. However, domains such as medication explanation and quietness, despite low appreciation from patients, are not as often investigated as communication measures due to their weaker relationships to overall rating [6, 12]. Hospital characteristics are known to influence the evaluation of domains. For example, overall rating and communication receive higher scores by small hospitals than do large hospitals [6, 12]. As to the influence of hospital structural measures on the relationships among the domains, analyses through traditional methodologies is inherently restricted as previously noted.

In contrast, distance-based analysis, such as clustering, correspondence analysis and multidimensional scaling (MDS), simultaneously analyzes relationships among patient-satisfaction domains, revealing patterns in datasets. Specific data distribution is not required, and it is possible to analyze all domains simultaneously without focusing on specific variables, giving equal weight to underrated domains, global evaluation and communication measures alike. Such methods do not produce the same number of results as the domains. The distances are presented visually but the interpretation of the patterns based on similarities and dissimilarities is rather subjective, as they do not produce results such as provability calculated in inferential statistics. In this study, results of the three methods were taken into consideration when interpreting their patterns against a lack of objectivity. The methods have been applied in business and medicine, yet studies on patient satisfaction using these methods remain scarce [16, 19–22], and moreover, have not to our knowledge been used to compare the structure of patient satisfaction by patient and hospital structural measures except in our previous studies [26, 27]. Many studies apply one method, namely clustering, correspondence analysis or MDS, of which clustering has been most widely used. Yet certain studies attempt to cluster patients and not domains [16, 22, 28], while one study used multiple correspondence analysis [22]. Studies in the 1980s used MDS to sample patients [19–21]. The present study is designed to evaluate similarities among measures of patient satisfaction and to identify homogenous groups by three distance-based analysis methods for robustness, and to investigate hospital structural measures for influencing factors.

## Methods

### Data sources

The Hospital Consumer Assessment of Healthcare Providers and Systems (HCAHPS) survey [29–31] was the first nation-wide, standardized survey of patients’ perspectives of hospital care in the United States. The survey, containing 32 items measuring patients’ perceptions of their hospital experiences, was administered to a random sample of adult inpatients between 48 hours and 6 weeks after discharge. HCAHPS survey data used in this study were collected from July 2012 to July 2013, as were hospital structural datasets from the official Hospital Compare site (https://data.medicare.gov/data/hospital-compare) [31]. The survey results were reported as adjusted percentages of dichotomous data or a three-point scale for each hospital [31]. Many studies evaluating HCAHPS data use the “top box (High)”, the most positive response to the measures [6, 17, 29], whereas the present study uses all the data, minimizing the dependence on positive data, because the proportions of “Low” responses increase exponentially as survey response rates decrease [26].

### Hospital selection and hospital structural measures

The criterion used in our previous study were applied [26]. Hospitals selected for analysis were those: (1) whose survey response size was 50 and above, given that data with fewer than 50 responses may be too limited for reliable assessment of hospital performance, (2) who submitted completed patient surveys, (3) submitted survey response rates, and (4) whose data showed no discrepancies [31].

Structural characteristics selected for analysis were: (1) hospitals with survey response sizes (SRSs) of 50–99, 100–299, and ≥ 300 subjects, (2) acute care hospitals (ACHs) and critical access hospitals (CAHs), and (3) whether hospitals were registered in the systematic clinical database for cardiac/general surgery and for nursing/stroke care. Such registered hospitals submit sets of process and outcome data to government agencies.

### Patient survey measures

The survey had eight domains of care and two global evaluation measures [31]. Of these 10 measures, nine reported on a three-point scale were selected to produce contingency tables of measures and ratings. These nine measures included: (1) How often did nurses communicate well with patients? (nurse communication); (2) How often did doctors communicate well with patients? (doctor communication); (3) How often did patients receive help quickly from hospital staff members? (staff responsiveness); (4) How often was patients’ pain well controlled? (pain management); (5) How often did staff explain about medicines before giving them to patients? (medication explanation); (6) How often were patients’ rooms and bathrooms cleaned? (cleanliness); (7) How often was the area around patients’ rooms kept quiet at night? (quietness); (8) How did patients rate the hospital overall? (overall rating); and (9) Would patients recommend the hospital to friends and family? (hospital recommendation). The ratings on composite questions 1–5 and single questions 6–7 were scored as “always (High)”, “usually (Medium)”, or “sometimes/never (Low)”. Questions 8–9 are global evaluation measures. Responses to Question 8 were scored on a rating scale of 1–10 scale, with scores of 10–9 categorized as “High”, 8–7 as “Medium” and 6–0 as “Low”. Responses to Question 9 were categorized as “definitely yes (High)”, “probably yes (Medium)” and “probably/definitely no (Low)”. Question 10 on the patient survey (“Were patients given information about what to do during their recovery at home?”) was excluded from analysis due to its dichotomous nature.

### Analysis

**Fig. 1:**
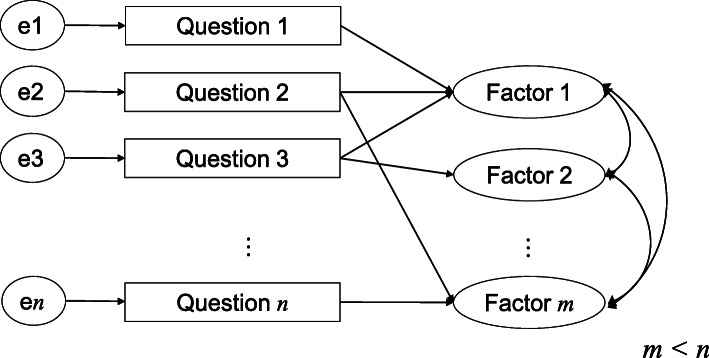
Model utilized to assess factors associated with patient satisfaction This model is for future work to assess the structure of patient satisfaction based on distance-based analysis. In this study the measures in the HCAHPS survey were used as surrogates for questions in the model and hospital structural measures were investigated for candidate factors. Arrows from questions to the same factors indicate they form a group. For example, questions 1, 2, 3 form a group by factor 1. Latency factors of this model are currently being investigated

We provide the model for future work to assess the structure of patient satisfaction according to distance-based analysis. In this study the measures in HCAHPS survey were used as surrogates for questions in the model, and hospital structural measures were investigated for candidate factors (see Fig. 1, which shows the model). In Fig. 1 arrows from questions to the same factors indicate they form a group. For example, question 1,2,3 form a group by factor 1. To explore the similarities of these measures and candidate factors, multiple-proportion tests (*p* < .05) were utilized to compare the proportions of hospitals with each characteristic with respect to SRSs as *a priori* surrogates for hospital size. The percentages of each measure were aggregated to produce contingency tables of the measures and the ratings of “High”, “Medium” and “Low” with respect to hospital structural measures. Subsequently, similarities among the measures were investigated by three analytic methods - clustering, correspondence analysis and MDS - which computed different distances for robustness based on the contingency tables.

Ward’s clustering method [32], an agglomerative hierarchical clustering procedure, was utilized to arrange the measures into homogeneous groups (clusters). This method calculates Euclidean distance and produces a two-dimensional diagram known as a dendrogram, in which similar measures merge at low heights and measures more dissimilar merge at higher points. The greater the height differences, the more dissimilar are the services. Large changes in a dendrogram may indicate a particular number of clusters [33]. Measures in a mutually exclusive cluster show that they were evaluated by similar patterns on the three-point scale.

Correspondence analysis was utilized to visualize the associations among rows (the measures) and columns (the ratings) in a contingency table simultaneously in scatterplots. Column points that are close together indicate columns with similar profiles, and row points that are close to column points represent combinations that occur more frequently [34]. The correspondence analysis method calculates chi-square distance and searches the axes (usually, two axes) which maximize the correlation ratio, and the sum of the squared correlation ratio of each axis is called an eigenvalue, denoted by *η*_1_^2^ and *η*_2_^2^ for the first (horizontal) and the second (vertical) axes, respectively. The contribution ratio measures the degree of which the axis obtained explains the nature of data, denoted by *γ*_1_ and *γ*_2_ for the first and the second axes, respectively. They are computed by dividing each eigenvalue by the total sum of eigenvalues.

Nonmetric MDS, a rank-based approach, was utilized to visualize the similarities among the measures in scatterplots based on a distance or dissimilarity matrix. This method calculates Euclidean distance. The lesser the similarity, the further apart the points representing them should be in the final geometrical model [35]. Also, the measures around the coordinate origin display proximities, and the measures on the periphery display dissimilarities.

All statistical analyses were performed using R software, version 3.1.0.

## Results

### Hospital structural measures

Of 4,677 hospitals, 3,711 (79.3%) met the inclusion criteria and were analyzed. Of these 3,711 hospitals, 220 (6%), 692 (19%), and 2,799 (75%) reported SRSs of 50–99, 100–299, and ≥ 300 patients, respectively. With respect to these three categories of SRSs, 78 (36%), 358 (52%), and 2,709 (97%) hospitals, respectively, were ACHs; 0 (0%), 8 (1%), and 1,005 (36%), respectively, were included in the cardiac surgery registry; 3 (1%), 27 (4%), and 590 (21%), respectively, were included in the general surgery registry; 5 (2%), 71 (10%), and 1,505 (54%), respectively, were included in the nursing care registry; 11 (5%), 54 (8%), and 1,518 (54%), respectively, were included in the stroke care registry; and 74 (34%), 288 (42%), and 1,790 (64%) respectively, used electronic health records (EHRs). Except for EHR, the percentage of each characteristic was significantly lower for hospitals with SRSs < 300 than ≥ 300 patients (p < .001 for nursing care; p < .0001) for all other factors [26]. Therefore, hospitals with smaller SRSs were integrated. These results substantiated our *a priori* use of SRSs as appropriate surrogates for hospital size [31].

### Similarities among hospital services by hospital structural measures

Analyses using distances were utilized to visualize the similarities and to identify natural groups among hospital services with respect to hospital structural measures. We first describe the results of hospitals that were and were not included in the cardiac surgery registry as they represent the features of our analyses in the order of clustering, correspondence analysis and MDS. Subsequently, we describe the results of other hospital structural measures.

**Fig. 2:**
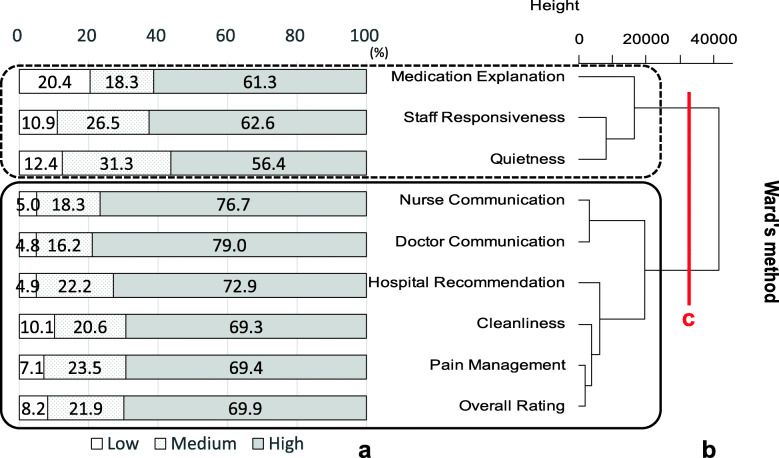
Clustering results of hospitals performing cardiac surgery *n* = 1013 “a” shows bar graphs of the proportions of each rating of the accumulated percentages by measures and “b” shows the dendrogram resulting from clustering of hospitals that performed cardiac surgery, with the rectangles indicating clusters. Numbers and a line at the top right indicate “Height,” which is distance. Large changes in height might be taken to indicate a particular number of clusters. Line “c” in Fig. 2 shows where to “cut” the dendrogram, indicating these hospitals performing cardiac surgery formed two clusters, with a better-rated cluster including doctor communication, nurse communication, pain management, overall rating, hospital recommendation and cleanliness, and a poorly rated cluster including medication explanation, staff responsiveness and quietness Revised from Fig. 3 in Okuda M, Yasuda A, Tsumoto S. Factors of Patient Satisfaction based on distant analysis in HCAHPS Databases, IEEE International Workshop on Data Mining for Service (DMS2014), Shenzhen, China, December 14, 2014 [36]

**Fig. 3:**
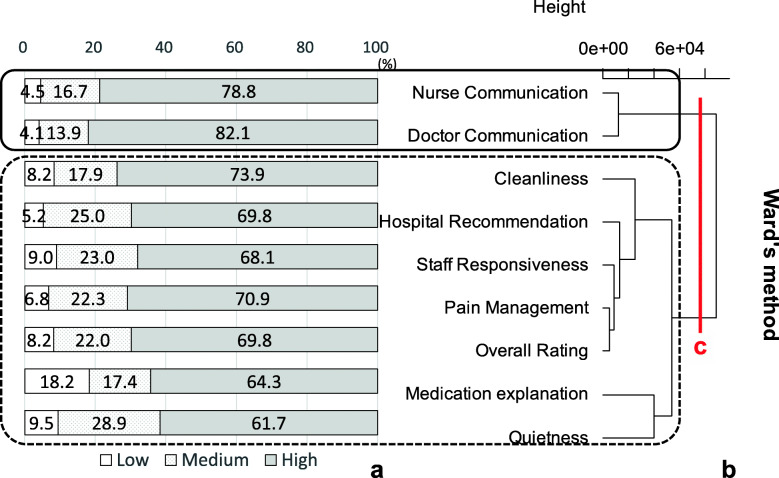
Clustering results of hospitals not performing cardiac surgery *n* = 2698 “a” shows bar graphs of the accumulated percentages by measures and “b” shows the clustering results, with the rectangles indicating clusters. “Height” at the top right means distance. Large changes in height were taken to indicate two clusters. Line “c” in Fig. 3 shows where to “cut” the dendrogram, indicating hospitals not performing cardiac surgery formed two clusters. Communication measures formed a better-rated group and the rest of the measures including two global measures formed a poorly-rated cluster Revised from Fig. 4 in Okuda M, Yasuda A, Tsumoto S. Factors of Patient Satisfaction based on distant analysis in HCAHPS Databases, IEEE International Workshop on Data Mining for Service (DMS2014), Shenzhen, China, December 14, 2014 [36]

In Fig. 2, “a” shows bar graphs of the proportions of each rating of the accumulated percentages by measures and “b” shows the dendrogram, resulting from clustering of hospitals that performed cardiac surgery, with the rectangles indicating clusters. In the dendrogram numbers and the line at the top right indicate “Height,” which is distance. Large changes in height might be taken to indicate a particular number of clusters [33]. Line “c” in Fig. 2 shows where to “cut” the dendrogram, indicating these hospitals performing cardiac surgery formed two clusters, a better-rated cluster including doctor communication, nurse communication, pain management, overall rating, hospital recommendation and cleanliness, and a poorly rated cluster including medication explanation, staff responsiveness and quietness. Figure 3 displays the results for hospitals that did not perform cardiac surgery. These hospitals also produced two clusters consisting of different combinations, with a better-rated cluster including doctor communication and nurse communication, and a poorly rated cluster including all other measures. However, the assesment of the clusters was rather subjective, based on descriptive statistics.

**Fig. 4:**
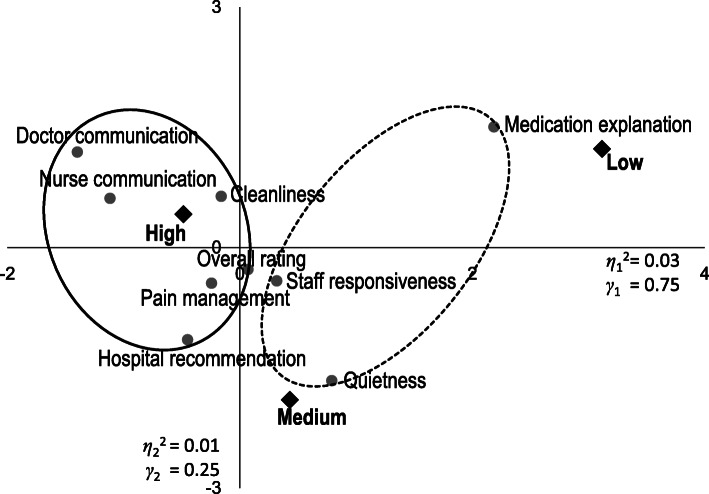
Results of correspondence analysis of hospitals performing cardiac surgery *n* = 1013 Fig. 4 illustrates the results of correspondence analysis of hospitals that performed cardiac surgery. Measures that are close together indicate measures with similar ratings and measures that are close to ratings represent combinations that occur more frequently. η_1_^2^ and η_2_^2^ near the axes denote the eigenvalues of the first (horizontal) axis and the second (vertical) axis respectively. The total sum of the eigenvalues 0.04 = 0.03 + 0.01. The contribution ratio of the first axis, γ_1_ 0.75 = 0.03/0.04 and of the second axis, γ_2_ 0.25 = 0.01/0.04, which means the first axis explains 75% of the variance in the data and the second 25%. In other words, the first two axes account for 100% of the variance of the data. The circles correspond to the clusters in Fig. 2. Measures in the better-rated cluster of doctor communication, nurse communication, pain management, cleanliness and two global evaluation measures were placed closely around “High” and far from “Medium” and “Low,” indicating the six measues received relatively similar better scores. In the other poorly rated cluster, quietness and staff responsiveness were the closest to “Medium” and medication explanation the closest to “Low” Revised from Fig. 3 in Okuda M, Yasuda A, Tsumoto S. Factors of Patient Satisfaction based on distant analysis in HCAHPS Databases, IEEE International Workshop on Data Mining for Service (DMS2014), Shenzhen, China, December 14, 2014 [36]

**Fig. 5:**
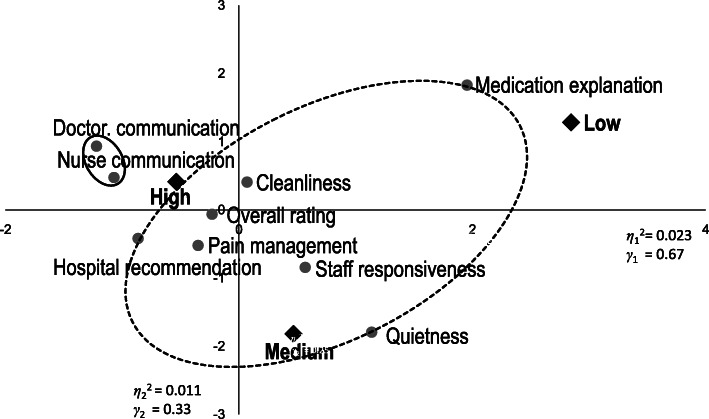
Results of correspondence analysis of hospitals that did not perform cardiac surgery. Figure 5 illustrates the results of correspondence analysis of hospitals that did not perform cardiac surgery. Measures that are close together indicate measures with similar ratings and measures that are close to ratings represent combinations that occur more frequently. η_1_^2^ and η_2_^2^ near the axes denote the eigenvalues of the first (horizontal) axis and the second (vertical) axis respectively. The total sum of the eigenvalues are 0.034 = 0.023 + 0.011. The contribution ratio of the first axis, γ_1_ is 0.67 = 0.023/0.034 and of the second axis, γ_2_ is 0.33 = 0.011/0.034, which means the first axis explains 67% of the variance in the data and the second 33%. In other words, the the first two axes account for 100% of the variance of the data. The circles correspond to the clusters in Fig. 3. Communication measures in the left circle were placed close to each other around “High” and far away from “Medium” and “Low,” indicating that scores were higher for communication measures than for the other seven measures in the right circle that are closer to “Medium” or “Low. Revised from Fig. 4 in Okuda M, Yasuda A, Tsumoto S. Factors of Patient Satisfaction based on distant analysis in HCAHPS Databases, IEEE International Workshop on Data Mining for Service (DMS2014), Shenzhen, China, December 14, 2014 [36]

Next, to investigate the relationships between the detected clusters and ratings, correspondence analysis was performed. Figure 4 illustrates the results of correspondence analysis of hospitals that performed cardiac surgery. Measures that are close together indicate measures with similar ratings, and measures that are close to ratings represent combinations that occur more frequently. In Fig. 4, *η*_1_^2^ and *η*_2_^2^ near the axes denote the eigenvalues of the first (horizontal) axis and the second (vertical) axis, respectively. The total sum of the eigenvalues 0.04 = 0.03 + 0.01. The contribution ratio of the first axis, *γ*_1_ 0.75 = 0.03/0.04 and of the second axis, *γ*_2_ 0.25 = 0.01/0.04, which means the first axis explains 75% of the variance in the data and the second 25%. In other words, the the first two axes account for 100% of the variance of the data. The circles in Fig. 4 correspond to the clusters in Fig. 2. Measures in the cluster of doctor communication, nurse communication, pain management, cleanliness and two global evaluation measures were placed closely around “High” and far from “Medium” and “Low.” In the other cluster, quietness and staff responsiveness were the closest to “Medium” and medication explanation the closest to “Low”. Figure 5 shows that the results of correspondence analysis for hospitals not performing cardiac surgery were somewhat different. The circles in Fig. 5 correspond to the clusters in Fig. 3. The communication measures were placed outside “High,” away from the other ratings and measures, whereas most of the measures in the other cluster, including overall rating, were between “High” and “Medium.” However, quietness and medication explanation were placed close to “Medium” and “Low”, respectively, similar to hospitals that performed cardiac surgery. The results of correspondence analysis statistically supported the clustering results, the evaluation differences between the clusters. Yet, in the graph of hospitals performing cardiac surgery, overall rating and pain management in the better-rated cluster and staff reponsiveness in the poorly rated cluster look closely-placed, as do communication measures and hospital recommendation in the graph of hospitals not performing surgery.

**Fig. 6:**
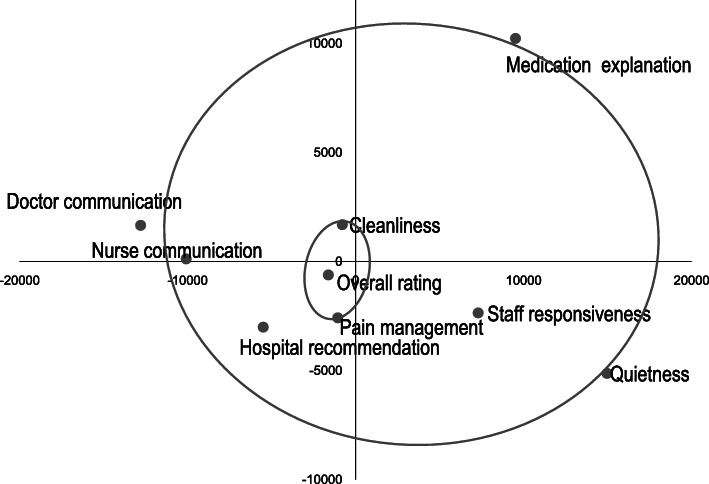
Results of multidimensional scaling of hospitals performing cardiac surgery *n* = 1013 In a scatter plot of MDS the lesser the similarity, the further apart the points (measures). Also, the measures around the coordinate origin display proximities, and the measures on the periphery display dissimilarities. Overall rating, pain management and cleanliness in the inner circle were located near the coodinate origin, whereas communication measures, quietness, and medication explanation were placed apart at the periphery indicated by the outer circle. Hospital recommendation and staff responsiveness were located midway between the measures near the coodinate origin and the periphery Revised from Fig. 5 in Okuda M, Yasuda A, Tsumoto S. Factors of Patient Satisfaction based on distant analysis in HCAHPS Databases, IEEE International Workshop on Data Mining for Service (DMS2014), Shenzhen, China, December 14, 2014 [36]

**Fig. 7:**
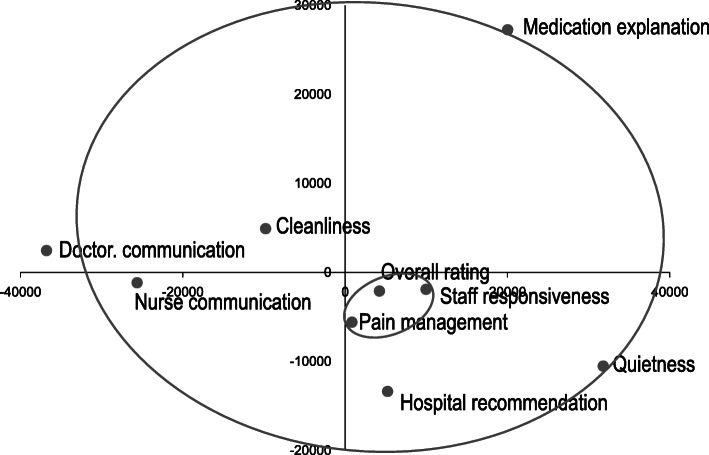
Results of multidimensional scaling of hospitals not performing cardiac surgery *n* = 2698 In a scatter plot of MDS, the lesser the similarity, the further apart the measures. Also, the measures around the coordinate origin display proximities, and the measures on the periphery display dissimilarities. Overall rating, pain management and staff responsiveness in the inner circle were located near the coodinate origin, whereas communication measures, quietness, and medication explanation were placed apart at the periphery indicated by the outer circle. Cleanliness was located midway between the measures near the coodinate origin and the periphery Revised from Fig. 5 in Okuda M, Yasuda A, Tsumoto S.Factors of Patient Satisfaction based on distant analysis in HCAHPS Databases, IEEE International Workshop on Data Mining for Service (DMS2014), Shenzhen, China, December 14, 2014 [36]

To examine the similarities and dissimilarities among the services, MDS was then performed. Figure 6 shows the results of MDS of hospitals that performed cardiac surgery, results similar to those of hospitals that did not (see Fig. 7). In a scatter plot of MDS, the lesser the similarity, the further apart the points. Also, the points around the coordinate origin display proximities, and the points on the periphery display dissimilarities. The results of both of the hospitals showed that overall rating and pain management in the inner circle were located near the coodinate origin, whereas communication measures, quietness, and medication explanation were placed apart at the periphery indicated by the outer circle, and that hospital reccomendation was located midway between the measures near the origin and the periphery. The differences between the hospitals were the measures located midway: staff responsiveness for hospitals that performed cardiac surgery and cleanliness for hospitals that did not. As to the seemingly closely-placed services, according to the results of correspondence analysis, they were in fact distanced. The results of MDS verified the results of clustering, indicating that medication explanation, quietness and the communication services were dissimilar from the other services.

Table 1 describes all the results of the analyses with respect to hospital structural measures. Each hospital characteristic showed two clusters, with open and closed shapes indicating higher- and lower-scored clusters, respectively. Open shapes “◎” and “〇” indicate measures included in a better-rated group, but “〇” suggest the possibility of forming another group in another study [26]. Closed shapes “▲”, “■” and “●” indicate measures included in a poorly rated group. Their shape differences suggest the possibilities of forming other groups, according to the results of correspondence analysis and MDS. For example, at all the hospitals two communication measures formed a better-rated group, suggested by “◎” shape, and also at all hospitals staff responsiveness, quietness and medication explanation formed a poorly rated group suggested by the closed shapes. However, cleanliness, pain management, overall rating and hospital recommendation show both of the open and closed shapes, suggesting patients’ evaluations were influenced by certain hospital structural measures.

**Table 1:** Results of clustering analysis, correspondence analysis and multidimensional scaling with respect to hospital structural measures.

Hospital Characteristics		*n*	Doctor communi-cation	Nurse communi-cation	Clean-liness	Pain management	Overall rating	Hospital recommendation	Staff responsiveness	Quiet-ness	Medication explanation
Survey response size 50–299		912	◎	◎	○	▲	▲	▲	▲	■	●
Survey response size 300~		2799	◎	◎	○	○	○	○	▲	■	●
Acute care hospital		3145	◎	◎	▲	▲	▲	▲	▲	■	●
Critical access hospital		566	◎	◎	◎	▲	▲	▲	▲	■	●
Electronic health record usage	Yes	2142	◎	◎	▲	▲	▲	▲	▲	■	●
	No	1569	◎	◎	▲	▲	▲	▲	▲	■	●
Cardiac surgery	Yes	1013	◎	◎	◎	◎	◎	◎	▲	■	●
	No	2698	◎	◎	▲	▲	▲	▲	▲	■	●
General surgery	Yes	620	◎	◎	◎	◎	◎	◎	▲	■	●
	No	3091	◎	◎	▲	▲	▲	▲	▲	■	●
Nursing care	Yes	1581	◎	◎	◎	◎	◎	◎	▲	■	●
	No	2130	◎	◎	◎	▲	▲	▲	▲	■	●
Stroke care	Yes	1583	◎	◎	◎	◎	◎	◎	▲	■	●
	No	2128	◎	◎	◎	▲	▲	▲	▲	■	●

*n* = 3711 Table 1 describes all the results of the analyses with respect to hospital structural measures. Each hospital characteristic showed two clusters, with open and closed shapes indicating higher- and lower-scored clusters, respectively. Open shapes “◎” and “〇” indicates measures included in a better-rated group. Closed shapes “▲”, “■” and “●” indicate measures included in a poorly rated group. Their shape differences suggest the possibilities of forming different groups, according to the results of correspondence analysis and MDS. For example, at all the hospitals two communication measures formed a better-rated group, suggested by “◎” shape and also at all hospitals staff responsiveness, quietness and medication explanation formed a poorly rated group suggested by closed shapes. However, cleanliness, pain management, overall rating and hospital recommendation show both of the open and closed shapes, suggesting hospital structural measures changed their ratings

Differences in shape within a cluster indicate possible dissimilarities between the results of correspondence analysis and MDS. Results for hospitals belonging to the cardiac surgery registry were similar to those for hospitals with SRS ≥ 300, to hospitals belonging to the general surgery nursing care, and to stroke care registries. Hospitals not belonging to the cardiac surgery registry exhibited the same clustering results as ACHs, as hospitals not belonging to the general surgery registry, and as hospitals that did and did not use EHRs. Cleanliness belonged to the communication cluster at hospitals with smaller SRS, CAHs, and at hospitals not belonging to the nursing care and stroke care registries. Correspondence analysis and MDS showed that cleanliness was closer to both communication measures than to the two global evaluation and pain management measures. While close examination in the previous study has shown that hospitals with SRS ≥ 300 display four clusters [26], for the sake of simplicity in this study we combined the four into two and evaluated them as two clusters.

## Discussion

### Similarities among measures of patient satisfaction

Based on the results of the three different analyses using distances, this study indicates that some hospital structural measures do not change the similarities of domains among patient satisfaction even though the same characteristics influence individual score differences within traditional analyses. In the present study, the nine HCAHPS measures were divided into three groups plus cleanliness, one group including doctor and nurse communications; a second group included pain management and two global evaluation measures; and a third group included medication explanation, quietness and staff responsiveness. Our study shows that hospital structural measures did not alter the similarities in these groups but did affect their combinations. This study also suggests dissimilarities among the measures with low scores (medication explanation, quietness and staff responsiveness) and dissimilarities between overall rating and hospital recommendation.

Doctor communication and nurse communication were similarly assessed at all hospitals, with more “High” scores than all other measures. Pain management and the two global evaluation measures received similar scores but were dependent on hospital structural measures. These three domains, together with doctor and nurse communication, formed the higher-rated group at hospitals having larger SRSs as well as in those on the cardiac/general surgery and nursing/stroke care registries. At the other hospitals, however, pain management and the two global evaluation measures joined medication explanation, quietness and staff responsiveness to form the lower-rated group.

Studies on communication with medical staff have yielded contradictory results, with some reporting better communication with nurse practitioners than with doctors [37], and others reporting better communication with doctors than with nurses [6, 12], and better communication with staff members at smaller than at larger hospitals [6, 12]. Our study, however, found that scores on communication with doctors and nurses were similar and relatively higher than other measures at all hospitals, independent of hospital structural measures. Patients likely appreciate human contact, regarding communication with health care personnel as a sign of respect and a tool to meet their care needs and for avoiding possible medical malpractice [38].

Although the overall rating shows a stronger correlation with nurse communication than with other measures including pain management [6, 9], our study found that the similarities between overall rating and nurse communication were not consistent, but were limited at large hospitals providing acute surgical treatment. This suggests that the value of communication of medical staff to patients differs according to a patient’s medical status. Patients in pain view communication as a verbal and attitudinal aspect of care, with scores similar to those of global evaluation, whereas patients in less pain not requiring specialized treatment were highly appreciative of communication with doctors and nurses but rated other hospital services as poor. This is likely an example of direct association among caring attitudes, swift pain treatment and patient satisfaction [15, 39].

Previous studies have reported that scores on medication explanation, quietness and staff responsiveness differ according to hospital structural measures [7, 13, 40]. However, our study found that these three measures received similar poor ratings at all hospitals. The backgrounds of these low evaluations differed, as correspondence analysis showed that medication explanation had a “Low” rating, quietness had a “Medium” rating and staff responsiveness had an intermediate rating. These results were supported by MDS, suggesting their dissimilarities. A qualitative study reported that differences in scores on staff responsiveness and quietness may be due to differences in patient expectations, as patients are more tolerant of slow responses than of hospital noise, as they seek a quiet environment [38]. Although medication explanation and communication measures would seem to be related, asking in the patient HCAHPS questionnaire if explanations were easy to understand [32], no similarities were shown. Our study indicates that providers’ efforts to explain medications to patients were insufficient, possibly due to patient worries about the possibility of serious side effects including death [38], and the doctors failing to fully describe a medication’s side effects [40].

Overall rating and hospital recommendation have been treated equally in patient-satisfaction studies. Although they show a strong correlation in HCAHPS studies [6], our study suggests their possible dissimilarities, as MDS analysis placed them at some distance from one another, indicating patients regarded overall rating and hospital recommendation as being distinct, requiring further investigation.

Cleanliness was one measure that differed among groups of hospitals. For example, ACHs and CAHs showed similar results, except for cleanliness, as did hospitals outside the cardiac/general surgery registries and those outside the nursing/stroke care registries. ACHs may be a mixture of the three categories of SRSs, as ACHs represent between one-third and one-half of hospitals with smaller SRSs. Hospitals outside the nursing/stroke care registries may be less able to control the quality of medical care or have fewer resources as they do not submit process and outcome data to the government. However, these results are not due solely to the attitude toward quality control, as cleanliness is not the only measure poorly rated at these hospitals [8, 11]. A qualitative study reported that patients regard lack of cleanliness as a possible indicator of infection [37] and bivariate analysis has shown relationships between cleanliness and technical quality [8, 11]. The reasons for differences in cleanliness among groups of hospitals require further investigation. While previous studies have reported mixed results on whether EHR usage influences patient satisfaction [17, 41], our study found that it did not.

### Candidate factors for the structure of patient satisfaction

The results of this study suggest that hospital size, hospital type, the ability to provide acute surgical treatment and hospital interest in improving the quality of medical care were factors that may influence the structure of patient satisfaction, whereas EHR usage was not. In future work to validate the model in Fig. 1 it would be necessary to prepare a patient satisfaction questionnaire including questions about the possible factors such as hospital size and patients’ health status for purposes of analyzing patient-level data. It would be also interesting to investigate, for example, whether medication explanation, quietness and staff responsiveness are similar, whether the dissimilarities of overall rating and hospital recommendation exist, and which patient and hospital structural measures would have influence on the structure of patient satisfaction.

### Study Limitations

It should be noted that our analyses utilized adjusted percentages of hospital-level data. Analyses of real numbers are easier to grasp but have the disadvantage of being more heavily influenced by larger numbers. In our study, over 75% of all hospitals had SRSs ≥ 300. Using percentages can avoid the disadvantages of data imbalances. However, whereas the questionnaire developed from the HCAHPS survey data compared differences between hospital-level and individual-level findings [42], our methods did not. Individual-level data may produce different results. Moreover, hospital-level data are not appropriate to validate the model since contingency tables of various patient characteristics and ratings would be necessary to validate the model.

There is also a possibility that since the survey data used in this study were collected from 2012 to 2013, analyses using the data in recent years may produce different results.

### Improving patient satisfaction

Through use of distance-based analysis, it should be possible to gain greater insight to understanding a patient’s view on hospital services. More attention should be paid to similarities among hospital services with respect to a patient’s background to better understand the depth of patient satisfaction.

This study using hospital-level data indicates hospitals should focus on medication explanation, noise reduction and rapid staff response, especially at large hospitals providing acute surgical treatment. At smaller hospitals, the improvement of pain management may lead to improvements in overall rating. Investigating the backgrounds of these groups will enhance the understanding of patients’ viewpoints and behavior, thereby improving the quality of medical care.

## Conclusions

This study sought to create a model by which to evaluate and assess through distance-based analysis changes in the structure of patient satisfaction with hospital structural measures in a publicly accessible dataset reported as hospital-level data, and to assess similarities among measures of patient satisfaction not possible by traditional analyses. Patients’ attitudes toward hospital services were sorted into three groups, one more highly rated consisting of aspects of communication with health care providers, a second with varying rating levels comprised of measures of global evaluation and pain management, and a third lower-rated group consisting of measures of medication explanation, quietness and staff responsiveness Cleanliness, alone, constituted a fourth group. High value ratings for communication and low value ratings for medication explanation, quietness and staff responsiveness were not influenced by hospital structural measures, but the varied-ratings domain group similarities, including items such as global evaluation and pain management, were affected by hospital structural measures.

This study suggests that hospital size, the ability to provide acute surgical treatment, and hospital interest in improving the quality of medical care were factors that may influence the structure of patient satisfaction. Analyses using distances helped reveal the hidden structure of patient satisfaction.

To validate the model, however, it will be necessary to analyze patient-level data. Further analyses of individual-level data, other structural data, processes and outcomes, and investigations of the factors underlying these results are needed to explore patient attitudes toward hospital services. These analyses can be applied to all studies using questionnaires.

## Data Availability

Available at Hospital Compare Data Archive [http://www.medicare.gov/download/HospitalCompare/2014/January/HOSArchive_Revised_Flatfiles_20140101.zip].

## Abbreviations

MDS: Multiple dimensional scaling
HCAHPS: The Hospital Consumer Assessment of Healthcare Providers and Systems
SRS: Survey response size
ACH: Acute care hospital
CAH: Critical access hospital
EHR: Electronic health record
